# Reasoning like a doctor or like a nurse? A systematic integrative review

**DOI:** 10.3389/fmed.2023.1017783

**Published:** 2023-03-03

**Authors:** Jettie Vreugdenhil, Sunia Somra, Hans Ket, Eugène J. F. M. Custers, Marcel E. Reinders, Jos Dobber, Rashmi A. Kusurkar

**Affiliations:** ^1^Research in Education, Amsterdam UMC Location Vrije Universiteit Amsterdam, Amsterdam, Netherlands; ^2^VUmc Amstel Academie, Institute for Education and Training, Amsterdam UMC Location Vrije Universiteit Amsterdam, Amsterdam, Netherlands; ^3^Faculty of Psychology and Education, LEARN! Research Institute for Learning and Education, Vrije Universiteit Amsterdam, Amsterdam, Netherlands; ^4^GGD Haaglanden, The Hague, Netherlands; ^5^Medical Library, Vrije Universiteit Amsterdam, Amsterdam, Netherlands; ^6^Faculty of Educational Sciences, Open Universiteit, Heerlen, Netherlands; ^7^Family Medicine, Amsterdam UMC Location Vrije Universiteit Amsterdam, Amsterdam, Netherlands; ^8^Center of Expertise Urban Vitality, Faculty of Health, Amsterdam School of Nursing, Amsterdam University of Applied Sciences, Amsterdam, Netherlands; ^9^Amsterdam Public Health, Quality of Care, Amsterdam, Netherlands

**Keywords:** clinical reasoning, nursing, medical, practitioners, layered analysis, concept analysis, interprofessional education

## Abstract

When physicians and nurses are looking at the same patient, they may not see the same picture. If assuming that the clinical reasoning of both professions is alike and ignoring possible differences, aspects essential for care can be overlooked. Understanding the multifaceted concept of clinical reasoning of both professions may provide insight into the nature and purpose of their practices and benefit patient care, education and research. We aimed to identify, compare and contrast the documented features of clinical reasoning of physicians and nurses through the lens of layered analysis and to conduct a simultaneous concept analysis. The protocol of this systematic integrative review was published doi: 10.1136/bmjopen-2021-049862. A comprehensive search was performed in four databases (PubMed, CINAHL, Psychinfo, and Web of Science) from 30th March 2020 to 27th May 2020. A total of 69 Empirical and theoretical journal articles about clinical reasoning of practitioners were included: 27 nursing, 37 medical, and five combining both perspectives. Two reviewers screened the identified papers for eligibility and assessed the quality of the methodologically diverse articles. We used an onion model, based on three layers: Philosophy, Principles, and Techniques to extract and organize the data. Commonalities and differences were identified on professional paradigms, theories, intentions, content, antecedents, attributes, outcomes, and contextual factors. The detected philosophical differences were located on a care-cure and subjective-objective continuum. We observed four principle contrasts: a broad or narrow focus, consideration of the patient as such or of the patient and his relatives, hypotheses to explain or to understand, and argumentation based on causality or association. In the technical layer a difference in the professional concepts of diagnosis and the degree of patient involvement in the reasoning process were perceived. Clinical reasoning can be analysed by breaking it down into layers, and the onion model resulted in detailed features. Subsequently insight was obtained in the differences between nursing and medical reasoning. The origin of these differences is in the philosophical layer (professional paradigms, intentions). This review can be used as a first step toward gaining a better understanding and collaboration in patient care, education and research across the nursing and medical professions.

## 1. Introduction

When physicians and nurses are looking at the same patient, they may not see the same picture (1). If clinicians assume that the clinical reasoning of different professions is alike, they may miss significant aspects and a more comprehensive picture of the patient situation (2–4). Yazdani and Hoseini Abardeh (5) characterize clinical reasoning as “a challenging, promising, complex, multidimensional, mostly invisible, and poorly understood process.” Clinical reasoning has been defined and studied “within” each profession. To date, it is unclear if the content, process, and outcomes are comparable “between” professions. In this review, we focused on the two largest healthcare disciplines (6), physicians and nurses, to explore this gap in the literature. Ignorance about differences might hamper collaboration in patient care, interprofessional education and even the transferability of research findings. Understanding the clinical reasoning approaches of both professions may provide insight into the nature and purpose of their practices. A common language for clinical reasoning might benefit communication, education, research, and patient care (2, 7).

Clinical reasoning is described as a multifaceted concept (8, 9) and as a complex concept for the literature uses many terms, which are either synonyms or related or surrogate terms (8, 10, 11). For the purpose of this paper we use the definition of Simmons (12), because it is used in medical and nursing literature: clinical reasoning is “a complex cognitive process that uses formal and informal thinking strategies to gather and analyse patient information, evaluate the significance of this information and weigh alternative actions.” Professionals use clinical reasoning to diagnose and to choose interventions or treatments; they practice either diagnostic or management (therapeutic) reasoning (13–15).

Comparing the clinical reasoning of professionals is challenging. Not only does clinical reasoning take place in the heads of individuals (16), differences have also been identified between novices and experienced and expert professionals (17, 18) and between doctors of different medical disciplines (19). Moreover, the reasoning of professionals seems to adjust to the complexity of each patient’s problem (17) and to the current context (20, 21). This flexibility aspect of clinical reasoning leads to a disunited view of the concept of clinical reasoning.

Differences between professions can be explained by their unique professional focus and knowledge, although clinical reasoning is more than operating on a knowledge base (9). Clinical reasoning can be studied from a cognitive, situated, linguistic or social perspective, (11) with the aim to explain either the process of reasoning, the knowledge structures or the cognitive modes (e.g., intuition or analysis) that are used (5). All these aspects have been investigated within the boundaries of the medical or nursing profession. A few studies have been carried out to investigate how both reasoning approaches relate to each other. To our knowledge, no systematic review of similarities and differences in the clinical reasoning of medical and nursing professionals has been published.

To do justice to the multifaceted nature of clinical reasoning, we aimed to compare and contrast “all” the facets of clinical reasoning in the medical and nursing literature. For this purpose we adapted and combined the model of layered analysis of educational interventions of Cianciolo and Regehr (22) and the concept analysis of Walker and Avant (23). Our intention was to “peel the shells of the clinical reasoning onion” in order to make this term accessible for analysis. Through the lens of layers and concepts, we aimed to answer the following research questions: what are the features of clinical reasoning of professional practitioners as described in medical and nursing scientific literature, and what can we learn about clinical reasoning from this simultaneous concept analysis? Our broader ambition is to improve mutual understanding and collaboration in patient care, education and research by increasing the conceptual transparency of clinical reasoning among nurses and physicians.

## 2. Methods

### 2.1. Protocol and registration

The protocol of this systematic integrative review was published in BMJ Open, doi: 10.1136/bmjopen-2021-049862 (24). After this publication, we further refined the layered analysis, which will be explained in the sub-section layers, shells, and cells.

### 2.2. Search strategy

We followed the criteria of the Preferred Reporting Items for Systematic Reviews and Meta-Analyses (PRISMA) 2020 statement (25). The search strategy was developed by JV and a clinical librarian (HK) and was carried out from 30 March 2020 to 27 May 2020. We searched in the databases Pubmed, CINAHL, Psychinfo, and Web of Science for methodological diverse articles on the clinical reasoning of nurses, physicians, or both, in all kinds of practice settings and specialties. The full search strategies for all databases are included in Appendix 1. Because of the high number of identified articles in this search, we purposefully restricted the sample to records from 2000 to May 2020 (26, 27). The underlying arguments were that from this date clinical reasoning was given a place in the professional competency sets (28–30), and reviews, based on older studies were not excluded in our strategy. To discover other studies relevant to the layers of our research question, we applied ancestry searching by screening the references of included studies (31, 32), also to ascertain that important earlier studies would not be missed.

### 2.3. Study selection

The records were downloaded into Rayyan and Endnote, and duplicates were removed. The titles and abstracts of the records were screened by JV and RK in Rayyan by applying the selection criteria agreed on by the full research team (Table 1).

**TABLE 1:** Selection criteria.

Criteria	Inclusion	Exclusion
Types of publication	Journal publications	Theses, dissertations, books, articles without abstract
Study population	Practicing physicians, nurses	Other health professionals, medical and nursing students, residents, non-practicing physicians and nurses, advanced nurse practitioners
Types of research	Quantitative, qualitative, empirical, theoretical, expert opinions, reviews	Case studies
Setting	Practice in all healthcare settings	In-school or university setting, simulation, training
Focus of article	Clinical reasoning, judgment, synonyms of reasoning and judgment, reasoning approaches and processes, comparison, collaborations of physicians and nurses, diagnostic uncertainty	Decision making (tools, decision making analysis), normative approaches, critical thinking, Bayesian thinking, intuition, education, educational interventions, assessment, accuracy of reasoning, moral reasoning
Publication period	Initially from Inception-May 2020, later restricted to 2000-May 2020	–

Differences in inclusion and exclusion decisions were discussed until agreement was reached. The full-text publications were loaded into Endnote and selected by one author (JV) (33), based on the established inclusion and exclusion criteria.

### 2.4. Quality assessment

JV and SS independently appraised the quality of the provisionally included studies with an instrument of Badu et al. (34) which fits methodologically diverse research reports, as described in our protocol. Assessment differences were small and discussed until agreement was reached.

### 2.5. Data extraction and processing in layers, shells, and cells

From the included papers, we extracted data according to the planned data items, i.e., the layers of clinical reasoning, which are summarized in Table 2.

**TABLE 2:** Layers and shells of clinical reasoning.

Onion	Layers	Onion shells	Description
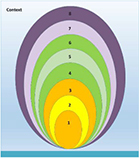	Techniques	Contextual factors (8)	Non-medical factors that influence the reasoning process and outcomes, such as characteristics of patients, health care system, and environment (20, 74)
		Outcomes (7)	Results of clinical reasoning, events that occur as a result of a concept, also referred to as consequences (23)
	Principles	Attributes (6) Antecedents (5)	The defining characteristics of a phenomenon, the core of a concept analysis (23); we used the categories of attributes of Cote (75) to define the cells of this shell Events, phenomena, behaviors, conditions, or attitudes (23) that precede clinical reasoning
		Content (4)	Data about the domain of reasoning, or what professionals reason about
	Philosophy	Intentions (3)	Information about goals which can describe reasoning as an entity with a stable identity or essence, even when adapted to other circumstances (22)
		Theories (2)	Internally consistent groups of relational statements about a phenomenon (23) that are used to describe, explain, or prescribe clinical reasoning. Guided by our research question, we limited ourselves to data about descriptive theories which indicate how professionals actually reason (12)
		Professional paradigm (1)	A constellation of shared beliefs, agreements, habits, language, and procedures (36, 76). These perceptions and expectations are the essence which goes beyond all other findings of clinical reasoning (4)

We used the three layers identified by Cianciolo and Regehr (22), philosophy, principles and techniques. These layers have blurry boundaries. Besides, the layers differ in their sensitivity to change under variable circumstances. The core layer, philosophy, includes underlying intentions, essence, and philosophies. To capture this layer, we searched for three types of data (text fragments or purports): professional paradigms, underpinning theories, and intentions or goals of clinical reasoning. Under the middle layer, principles, we grouped another three dimensions of clinical reasoning: the content, the antecedents, and the attributes; together, they reflect the structural aspects of clinical reasoning. Although the attributes also represent the techniques of reasoning, we added the attributes in the layer of principles under the assumption that they are less sensitive to change than the last two shells of the techniques layer: outcomes of reasoning and contextual factors. Under the shells, the data were clustered into cells.

JV and SS independently extracted the data from five studies, randomly chosen, to improve delineation of the layers and shells by discussing the (minor) differences. JV extracted the rest of the data into validity matrices (35), one for each shell, with columns for nursing and medicine, and clustered them into cells, i.e., categories of data elements. These data elements were the fourth tier of our data collection. The validity matrices were discussed in the full research team in several rounds of summarizing and reduction, to manage the large amount of data.

### 2.6. Patient and public involvement

Patients, students, and educators were included in this review only through inclusion of what was written about them in the published reports.

## 3. Results

### 3.1. Study selection

**FIGURE 1:**
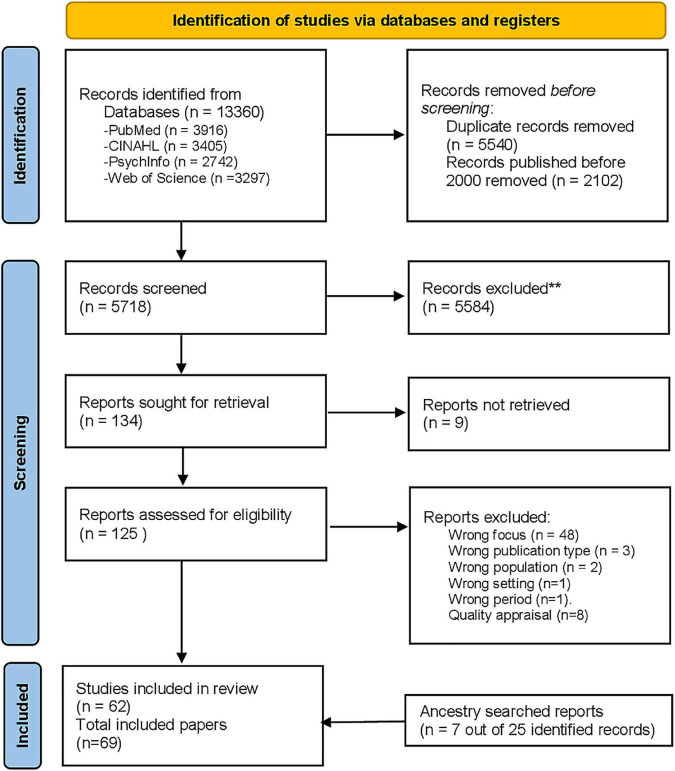
Preferred Reporting Items for Systematic Reviews and Meta-Analyses (PRISMA) 2020 flow diagram (25).

The search of four databases for papers about the clinical reasoning of physicians and nurses identified 5,718 unique records. Based on the screening of titles and abstracts with our selection criteria, we reviewed 125 full text reports, 55 of which were excluded because they did not fit the selection criteria. Eight papers were excluded during quality assessment (JV, SS) because of missing research questions or aims. Of the 24 records identified through ancestry searching, we included seven—mostly published before 2000–because of their relevance to one or more layers. The study selection is summarized in Figure 1.

### 3.2. Study characteristics

Of the selected reports, 27 studies reported on nursing research, 37 on medical research and five studies combined nursing and medical perspectives. The included reports used diverse methods: empirical and secondary studies, qualitative and quantitative studies, systematic reviews, concept analyses, and expert opinions. The selected studies, their study types and quality assessment ratings are presented in Table 3.

**TABLE 3:** Included studies.

References	Year	Type of study	Quality assessment	Profession
Adams et al. (56)	2016	Qualitative	88%	M
Alam et al. (77)	2017	Systematic review	91%	M
Austgard (43)	2008	Text, opinion, review	93%	N
Balla et al.(72)	2012	Qualitative	75%	M
Banning (57)	2008	Text, opinion, review	93%	N
Benbassat (44)	1996	Text, opinion, review	ancestry searched	M
Bissessur (14)	2009	Text, opinion, review	100%	M
Blondon et al. (39)	2017	Mixed methods	92%	MN
Bonilauri Ferreira et al. (51)	2010	Qualitative	82%	M
Buckingham et al. (59)	2000	Text, opinion, review	100%	N
Cader et al. (78)	2005	Text, opinion, review	93%	N
Cappelletti et al. (79)	2014	Systematic review	85%	N
Charlin et al. (80)	2012	Qualitative	ancestry searched	M
Charlin et al. (81)	2000	Text, opinion, review	100%	M
Chiffi et al. (53)	2015	Text, opinion, review	93%	MN
Cote et al. (75)	2012	Text, opinion, review	80%	N
Cox (82)	2002	Text, opinion, review	100%	M
Crook (37)	2001	Text, opinion, review	100%	N
Croskerry (62)	2009	Text, opinion, review	100%	M
Crow et al. (48)	1995	Text, opinion, review	ancestry searched	N
Davis (83)	1997	Text, opinion, review	ancestry searched	M
Dumas et al. (84)	2018	Text, opinion, review	100%	M
Durning et al. (63)	2012	Mixed methods	75%	M
Durning et al. (85)	2013	Text, opinion, review	100%	M
Edwards et al. (86)	2004	Quantitative, non-randomized	88%	M
Elstein et al. (87)	2002	Text, opinion, review	100%	M
Evans and Trotter (88)	2009	Quantitative, non-randomized	ancestry searched	M
Fawcett (52)	2010	Text, opinion, review	100%	N
Franco (36)	2014	Text, opinion, review	ancestry searched	M
Goldszmidt et al. (89)	2013	Quantitative, descriptive	100%	M
Groves et al. (90)	2003	Quantitative, non-randomized	78%	M
Gupta et al. (40)	2019	Text, opinion, review	100%	M
Holder (91)	2018	Systematic review	62%	N
Johnsen et al. (49)	2016	Qualitative	82%	N
Judd (92)	2005	Text, opinion, review	100%	N
Juma et al. (93)	2017	Qualitative	75%	M
Kiesewetter et al. (33)	2017	Systematic review	78%	M
Lee et al. (94)	2016	Qualitative	75%	N
Lee et al. (42)	2006	Text, opinion, review	100%	N
Levett-Jones et al. (95)	2010	Text, opinion, review	100%	N
Loftus (96)	2012	Text, opinion, review	100%	M
Malterud (41)	2002	Text, opinion, review	100%	M
Malterud et al. (73)	2019	Qualitative	87%	M
Marcum (97)	2013	Text, opinion, review	100%	M
McLean (98)	2017	Qualitative	100%	MN
Mirza et al. (38)	2014	Text, opinion, review	100%	N
Norman (69)	2005	Text, opinion, review	100%	M
Norman et al. (99)	2007	Text, opinion, review	100%	M
Passos Vaz da Costa et al. (60)	2016	Text, opinion, review	100%	N
Pelaccia et al. (55)	2015	Qualitative	82%	M
Pelaccia et al. (50)	2020	Text, opinion, review	100%	M
Pomeroy et al. (68)	2010	Mixed methods	68%	M
Pottier et al. (71)	2011	Text, opinion, review	100%	M
Psiuk (100)	1997	Text, opinion, review	ancestry searched	N
Quaresma et al. (101)	2019	Text, opinion, review	100%	N
Round (102)	2001	Text, opinion, review	93%	M
Salantera et al. (47)	2003	Quantitative, non-randomized	95%	MN
Shin (103)	2019	Text, opinion, review	100%	M
Simmons (12)	2010	Text, opinion, review	100%	N
Simmons et al. (58)	2003	Qualitative	82%	N
Stolper et al. (104)	2011	Text, opinion, review	100%	M
Tanner (54)	2006	Text, opinion, review	100%	N
Taylor (61)	2006	Qualitative	82%	MN
Twycross et al. (64)	2006	Qualitative	82%	N
van Graan et al. (105)	2016	Text, opinion, review	100%	N
Victor-Chmil (106)	2013	Text, opinion, review	100%	N
Yang et al. (107)	2014	Quantitative, non-randomized	82%	N
Yazdani et al. (74)	2017	Text, opinion, review	100%	M
Καρρά Β, et al. (108)	2018	Qualitative	88%	N

### 3.3. The layered data analysis: Shells, cells, and data elements

**FIGURE 2:**
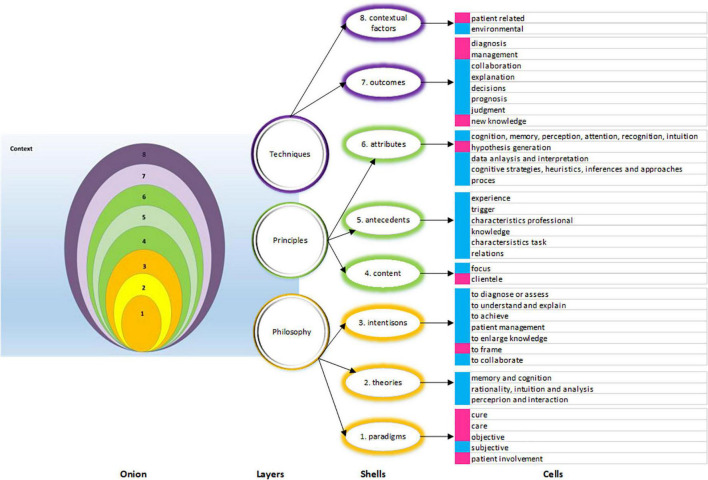
Overview of commonalities and dissimilarities found in the layers, shells and cells. Three layers (purple, yellow, green), eight shells, made up of cells. The cells marked in blue represent commonalities, the cells marked in pink show dissimilarities in reasoning.

We arranged all our findings (data elements or quotes) in validity matrices, clustered in shells and cells, as shown in Appendix 2. A rough overview of commonalities and dissimilarities found in our layered analysis is depicted in Figure 2.

#### 3.3.1. Philosophy: Paradigms

We classified the extracted data on paradigms into five cells. In the included studies, the nursing, and medical paradigms differ. The medical articles focused on the medical (curing) paradigm of diagnosis and treatment (36), while the nursing articles focused on a pragmatic paradigm with encompassed caring (37, 38). However, nursing studies also reported on nurses who play a role in diagnosis and treatment (39), while in medical literature, clinical care, and a functional health paradigm are mentioned as well (36, 40). Based on the literature, care as well as cure seems to be given attention by both professions, while the emphasis on either may differ. On an objective–subjective continuum, nurses and physicians both recognize subjectivity in their task perceptions, but they differ in their appreciation of objective “knowing” and in the degree to which they find it important. Medicine is often based on empirical knowledge, abstracted from the context and the patient (41), while nursing care is better described using a holistic view on the individual patient (37, 42, 43). By aggregating the data, we suggest that these differences as well as commonalities to be viewed on continuums, a care-cure continuum, and a subjective-objective continuum. The latter is substantiated in the most noticeable documented difference: in nursing, a patient is involved in clinical reasoning, while in medicine, is this not necessary (42–44).

#### 3.3.2. Philosophy: Theories

We found three types of theories in both the nursing literature and the medical literature: on memory and cognition, on rationality, analysis and intuition, and on perception and interaction. The following theories are prominent examples of these three types of theories:

- Information processing theories that aim to explain how perceived information is related to knowledge, knowledge storage, and retrieval from memory (45).
- Theories on analytic, conscious, slow, intuitive, implicit or fast thinking, either viewed as a dual process or on a continuum, based on the characteristics of the task (17).
- Situativity theory includes context and experience to explain thinking, learning, and knowledge (46).

In the medical literature, more theories are used to explain contextual influences, perception and interaction in particular, than in the nursing reports.

#### 3.3.3. Philosophy: Intentions

The intentions of clinical reasoning seem to be shared to a large extent by physicians and nurses. We categorized the data into seven cells: to diagnose or assess, for patient management (e.g., to decide on a plan of actions), to understand and explain, to enlarge knowledge, to collaborate, to achieve, and to frame (Appendix 2 and Figure 2). The differences observed at the cell level were related to the degree of autonomy or initiative: to establish (physician) or to recognize (nurse), to manage (physician) or to reduce (nurse), and to frame an encounter (physician). Moreover, physicians aim to diagnose and plan treatments, while nurses aim to reconstruct their understanding of the problems in a constantly changing situation (47, 48) and to understand symptoms and their impact on the patient (39, 49).

#### 3.3.4. Principles: Content

Much of the content of clinical reasoning is similar for physicians and nurses. However, physicians have a narrower focus, which is on the illness and its causes, while nurses have a broader focus, which is on the content or domain of their care. Besides feeling responsible for illness and health, nurses also feel responsible for the consequences of the patients’ health problems. It is a matter of the “sickness” or the “sick person.” Nonetheless, also in medicine, in acute situations, management of the patient’s condition precedes diagnosis of the disease (50). Bonilauri Ferreira et al. (51) found that physicians rely more on patient-specific heuristics than on disease-specific clinical guidelines. The most prominent difference is that physicians focus on an individual patient as the object of reasoning and that nurses may include the nearest and the dearest (the relatives) and even a patient’s community (47, 52–54).

#### 3.3.5. Principles: Antecedents

We grouped the aspects preceding clinical reasoning into professional experience, knowledge, triggers, task characteristics, a professional’s characteristics, and relations (Appendix 2 and Figure 2). Knowledge and experience are shared prerequisites of reasoning, although there is a difference between the topics of formal knowledge. Nurses tend to use more experiential knowledge, whereas physicians tend to use more theoretical knowledge (47). Concerning the triggers and task characteristics, nurses have a broader view (“life situation” versus “illness”). Physicians are triggered by contextual data, such as how a patient arrives at the Emergency Room (55). Nurses, alternatively, are triggered by the patient’s needs, for example “I could see today that she is low, she looked tired and things are telling on her” (21). Diagnostic uncertainty has been identified as a trigger for further reasoning for physicians (56), but is not mentioned in the selected nursing articles.

#### 3.3.6. Principles: Attributes

The findings on attributes of clinical reasoning are clustered into five groups (Appendix 2 and Figure 2). Many attributes are shared between physicians and nurses, e.g., they act quite alike in the use of cognition. Differences are found in hypothesis formulation. Nurses’ hypotheses are aimed to explain or understand patient symptoms and often lack causality or predictive power. For example, nurses associate nausea with a medical treatment, they do not use physiological arguments. In medicine, a hypothesis can be justified by cause-and-effect arguments (37, 53). Analytic strategies for hypothesizing, which are often used by physicians, can be abstract and decontextualized (40), whereas we could not find these strategies in the nursing literature. Nurses use analytic strategies to classify and to link cues to categories (49, 57–59).

#### 3.3.7. Techniques: Outcomes

For both physicians and nurses, clinical reasoning leads to diagnosis, judgments, decisions, management plans, prognosis, explanations, collaboration, and new knowledge. However, nursing diagnoses differ from medical diagnoses. The aim of a nursing diagnosis is to identify the current situation, the responses to health problems of a patient and his relatives (48, 60). Since these responses or situations are variable, nursing diagnosis is an ongoing process to detect changes in the patient’s condition. By contrast, a medical diagnosis is made at a discrete time point and is relatively stable (48). According to Chiffi and Zanotti (53), the purpose of a medical diagnosis is to identify biological alterations, organic or functional, while for nurses it is to identify possibilities to enhance self-care. The importance of causality is more prominent in a medical diagnosis, while nursing diagnoses are often descriptive generalizations which are associated with a health problem. A medical diagnosis can be established without direct involvement of the patient, while this is often not possible in nursing, where the patient’s (or his or her relative’s) perception of their condition and their level of self-care are indispensable factors in formulating a diagnosis (43, 53). While patient management is a shared outcome, the physician designs the treatment plan that fits with the illness, while the nurse designs the care plan and chooses actions that fit with the patient’s condition, the medical treatment plan and the patient’s self-care goals. The two plans come together in the evaluation of parameters, of “the look” of the patient, and of the progress that has been achieved (61).

#### 3.3.8. Techniques: Contextual factors

The influence of environmental factors on clinical reasoning has been described in many nursing and medical articles (12, 33, 37, 50, 54, 62, 63). Some authors mentioned the characteristics of the professionals as contextual factors, whereas we chose to regard them as antecedents of clinical reasoning. The included reports differed in the labeling of patient-related factors. For instance, in studies on the clinical reasoning of physicians, they were regarded as contextual factors, whereas studies on the clinical reasoning of nurses regarded patient-related factors as part of the problem. This difference in labeling is related to our findings about paradigm, content, and outcomes, which indicated that nurses give their patients a different role in the reasoning process than physicians.

## 4. Discussion

In this systematic integrative review, we aimed to provide an overview of the commonalities and differences in the clinical reasoning of physicians and nurses by scrutinizing the data of the included studies with a detailed layered analysis, which resulted in our onion model. By breaking down the concept of clinical reasoning into layers, shells and cells, we were able to provide insight into these differences and commonalities. By comparing multiple facets of the clinical reasoning of these two professions, the content of clinical reasoning and the contrasts between medicine and nursing became clearer.

The main differences were found in the philosophical layer, where nurses and physicians were shown to have dissimilar professional paradigms considering the two continuums care-cure and objectivity-subjectivity and considering patient involvement, and where they used different professional expressions indicating more or less autonomy and more or less initiative. In the layer of principles, our results revealed four contrasts: a broader versus a narrower focus, consideration of the patient alone versus consideration of the patient and his relatives, the use of hypotheses for scientific explanation versus for holistic understanding, and argumentation based on causality versus argumentation based on association. The most notable differences between nurses’ and physicians’ clinical reasoning are the dissimilar concepts of diagnosis and the different usage of patient factors in the reasoning approach.

According to Chiffi and Zanotti (53) and Twycross and Powls (64), nurses need to know their patients and use their involvement to be able to reason about the required care. However, based on research on illness scripts (65, 66) we assume that the reasoning of nurses can also be triggered before patient acquaintance.

Part of the identified dissimilarities between the clinical reasoning of physicians and nurses can probably be explained by the fact that the majority of the records on medical clinical reasoning focused on diagnostic reasoning. If we compare our findings of nurses’ clinical reasoning with the characteristics of medical management reasoning or therapeutic reasoning, the differences become smaller. Compared to diagnostic reasoning, less is written about management reasoning of physicians. However, in patient care, management reasoning might be more relevant than diagnostic reasoning (13, 67). In management reasoning, the patients and their preferences are involved, the broader care situation is included, and ongoing monitoring and adjustment is required. While a diagnosis can be right or wrong, a management plan is chosen out of many options to fit the patient, the situation and the practitioner. Hence management reasoning, like nursing reasoning, is all about the dynamics, in time, between the players and the field.

A second finding is that most of the included studies focused on processes within individuals. Clinical reasoning is often described in terms of its attributes like cognition, memory, formal analysis, or intuition or in terms of the antecedents of knowledge and experience. These features are at the heart of the literature on clinical reasoning, and they mainly refer to individual processes. The process and content of clinical reasoning can vary between individuals (68) because individual experience may have more influence than training (50), and because a form of reasoning is used that fits the situation (57). The reasoning of professionals is also changeable due to time aspects. Professionals look at the present to identify events, at the past to identify causes and at the future to reason about prognoses and therapy (physicians and nurses) or about the patient’s future functioning (nurses) (53). The focus over the years on individual clinical reasoning aspects might have been chosen due to the complexity and multi-dimensionality of clinical reasoning (10). Moreover, this focus could be a result of the history of research on clinical reasoning on individual process, from problem solving to memory and mental representations, to the role of science and studies about non-analytic and analytic thinking (5, 69).

However, more importantly, in practice, the care of a patient usually involves more than one professional (33). In the context of interprofessional collaboration, more attention has been paid to the situative context of reasoning than to the individual processes. Terms like collaborative reasoning (33, 39) or ecological reasoning (70) are used to describe the sociological, environmental and team aspects of and influences on reasoning. Reasoning can be seen as a collaborative process (39, 71) and feedback is considered essential (72), as contradicting information from colleagues triggers further clinical reasoning (51, 57, 73). The existing differences between the reasoning of nurses and physicians can then be viewed as necessary and complementary (47). If both reasoning approaches are articulated and shared, the reasoning itself could be improved *via* debate (33, 59, 73), which can lead to an improved and more holistic picture of the patient (3, 39).

Education, research, and communication about clinical reasoning is complicated because of the “polyphony” in the terms, definitions and conceptualizations of clinical reasoning (10, 11, 16). With our findings on clinical reasoning, we can argue that it is worthwhile to pay attention to the layers, the onion shells and cells that make up the concept, and not to focus on clinical reasoning as a indivisible construct.

### 4.1. Strengths and limitations

The stepwise approach, grounded in guidelines and theoretical frameworks of layered analysis and concept analysis was developed to diminish bias and improve rigor. The extracted data were repeatedly discussed in the full research team to reach data reduction and organization and to debate the main differences. This approach might enhance the confidence in our findings. The use of an onion model to investigate and analyse a complex cognitive process increases the transparency of our results. An evident limitation of our study on the differences between the clinical reasoning of nurses and physicians is that we investigated what was written about their clinical reasoning in journal articles. We excluded oral reports or case studies, which may have told different, personal stories about clinical reasoning. We deliberately chose to use articles published in peer-reviewed journals because we assumed that they adequately reflect the current, depersonalized knowledge about clinical reasoning. The second limitation is inherent in the chosen method of an integrative review of methodologically diverse, empirical, and theoretical articles. Since we did not aim to evaluate evidence but to reach a more comprehensive understanding, we did not weigh data according to their evidential value but to their informational value. The third limitation is that we did not take cultural aspects into account. We did not exclude reports in languages other than English, but most of the included articles were written by European and North American researchers. Moreover, we did not check our findings for potential differences in the culture of hospitals, psychiatric institutions or home care, which could be considerable. These differences might be explored in future research. Finally, we limited our search to studies on clinical reasoning that were published in the last 20 years. However, in this subset, we did not place our findings on a chronological timeline to investigate changes in reasoning of physicians and nurses, which could also be a topic for future research.

### 4.2. Recommendations

Clinical reasoning is a multifaceted container concept. Our findings of the differences in facets of clinical reasoning, modeled in the onion, can be used in interprofessional teams in the clinics, as well as in clinical reasoning training programs for nurses and physicians, in interprofessional education and in research. If researchers or policy makers of one profession consider using the results of studies on the clinical reasoning of another profession, we recommend to not only check the used terms or definitions, but also to check the three layers philosophy, principles and techniques in order to decide if the evidence is meaningful for their research question.

Multidisciplinary collaboration can be improved based on the realization that differences in reasoning between professionals are facets of a shared concept (59). Like Salantera et.al. (47), we assume that the differences in reasoning described in our study must be cherished, since they may add value to patient care and to collaboration. Moreover, training professional nurses and physicians in understanding each other’s reasoning approach might contribute to better patient care.

## 5. Conclusion

We learned from the simultaneous analysis of clinical reasoning, that this complex and multidimensional concept can actually be analysed by breaking it down into layers. With our onion model of shells, cells, and data elements, we could identify the detailed features of clinical reasoning. Subsequently insight was obtained in the commonalities and differences in the reasoning of nurses and physicians. The origin of the differences is in the philosophical layer -professional paradigms and intentions-, which is in line with the model of layered analysis. The results of this review can be used as a first step toward gaining a better understanding and collaboration in patient care, education and research across the nursing and medical professions.

## Author contributions

JV and RK were responsible for the initial design of this study and selected eligible studies. JV and HK developed and executed the systematic search strategy. SS and JV ran the quality assessment and data extraction. JV conceptualized the review approach, the quality assessment and data extraction, and wrote the first and the final draft of the manuscript. JD, EC, MR, and RK contributed to the conceptualization of the method, data analysis and synthesis, and writing—review and editing of the manuscript. All authors contributed to the article and approved the submitted version.
