# Supplementary material for: Reasoning like a doctor or like a nurse? A systematic integrative review
Source: Front Med (Lausanne). 2023 Mar 3;10:1017783. doi: 10.3389/fmed.2023.1017783 (PMC10020202; doi:10.3389/fmed.2023.1017783)
Supplement: Supplementary file 1 [file Data_Sheet_1.pdf]

Resources

- PubMed
- CINAHL
- PsycInfo
- Clarivate Analytics/ Web of Science

Search strategies

PubMed March 30, 2020 (569)

Search	Query	Items found
#27	(#23 AND #26)	569
#33	(#32 NOT #27)	894
#32	(#31 AND #23)	927
#31	clinical judgement*[tiab] OR clinical judgment*[tiab]	7156
#30	(#29 NOT #27)	1434
#29	(#28 AND #23)	1461
#28	clinical decision making*[tiab]	16876
#26	clinical reason*[tiab]	3973
#24	(#22 AND #23)	6965
#23	"Nurses"[Mesh] OR "Nurse-Patient Relations"[Mesh] OR "Practice Patterns, Nurses'"[Mesh] OR "Schools, Nursing"[Mesh] OR "Nurse's Role"[Mesh] OR "Students, Nursing"[Mesh] OR "Nursing Assistants"[Mesh] OR "Societies, Nursing"[Mesh] OR "Nursing Stations"[Mesh] OR "Nursing"[Mesh] OR "nursing" [Subheading] OR "National Institute of Nursing ReU.S."[Mesh] OR "Nursing Informatics"[Mesh] OR "Models, Nursing"[Mesh] OR "Nursing Research"[Mesh] OR "Nursing Staff"[Mesh] OR "Education, Nursing"[Mesh] OR nurse[tiab] OR nurses[tiab] OR nursing*[tiab] OR jsubsetn	955248
#22	(clinical*[tiab] AND ("Decision Making"[Mesh] OR "Judgment"[Mesh])) OR clinical reason*[tiab] OR clinical judgement*[tiab] OR clinical judgment*[tiab] OR clinical decision*[tiab]	65710

Revision, PubMed April 15 2020 (1415 nurse, 2501 doctor)

(1 AND 2) OR (1 AND 3)

Search	Query	Items found
#44	#43 NOT #40	2501
#43	#36 AND #42	2722
#42	"Anesthetists"[Mesh] OR "Physicians"[Mesh] OR allergist*[tiab] OR anaesthesiologist*[tiab] OR anaesthesist*[tiab] OR anesthesiologist*[tiab] OR anesthesist*[tiab] OR cardiologist*[tiab] OR dermatologist*[tiab] OR doctor*[tiab] OR endocrinologist*[tiab] OR gastroenterologist*[tiab] OR general practitioner*[tiab] OR geriatrician*[tiab] OR geriatrist*[tiab] OR gerontologist*[tiab] OR gynaecologist*[tiab] OR gynecologist*[tiab] OR hepatologist*[tiab] OR hospitalist*[tiab] OR house staff*[tiab] OR	1132406

	intensivist*[tiab] OR intern[tiab] OR internist*[tiab] OR interns[tiab] OR internship*[tiab] OR medical speciali*[tiab] OR neonatologist*[tiab] OR nephrologist*[tiab] OR neurologist*[tiab] OR neurologist*[tiab] OR neurosurgeon*[tiab] OR obstetrician*[tiab] OR obstaetrician*[tiab] OR oncologist*[tiab] OR ophthalmologist*[tiab] OR orthopedist*[tiab] OR osteopath[tiab] OR osteopaths[tiab] OR otolaryngologist*[tiab] OR otologist*[tiab] OR paediatrician*[tiab] OR pathologist*[tiab] OR pediatrician*[tiab] OR physiatrist*[tiab] OR physician*[tiab] OR pulmonologist*[tiab] OR radiologist*[tiab] OR residencies[tiab] OR residency[tiab] OR resident*[tiab] OR resident[tiab] OR residents[tiab] OR rheumatologist*[tiab] OR surgeon*[tiab] OR urologist*[tiab]	
**#41**	#40 NOT *[569 PMIDs]*	**901**
**#40**	#36 AND #39	**1415**
**#39**	"Nurses"[Mesh] OR "Nurse-Patient Relations"[Mesh] OR "Practice Patterns, Nurses'"[Mesh] OR "Schools, Nursing"[Mesh] OR "Nurse's Role"[Mesh] OR "Students, Nursing"[Mesh] OR "Nursing Assistants"[Mesh] OR "Societies, Nursing"[Mesh] OR "Nursing Stations"[Mesh] OR "Nursing"[Mesh] OR "nursing" [Subheading] OR "National Institute of Nursing Research (U.S.)"[Mesh] OR "Nursing Informatics"[Mesh] OR "Models, Nursing"[Mesh] OR "Nursing Research"[Mesh] OR "Nursing Staff"[Mesh] OR "Education, Nursing"[Mesh] OR nurse[tiab] OR nurses[tiab] OR nursing*[tiab] OR jsubsetn	**956603**
**#36**	clinical reasoning*[tiab] OR clinical judgement*[tiab] OR clinical judgment*[tiab] OR collaborative reasoning*[tiab]	**10227**

Ebsco/CINAHL April 30, 2020 (2191 nurse, 1214 doctor)

#	Query	Results
**S8**	S6 NOT *[2501 PMIDs]*	**659**
**S7**	S3 NOT *[1415 PMIDs]*	**1,590**
**S6**	S5 NOT S3	**1,214**
**S5**	S1 AND S4	**1,436**
**S4**	MH "Physicians+" OR MH "Pathologists+" OR MH "Anesthetists" OR TI(allergist* OR anaesthesiologist* OR anaesthesist* OR anesthesiologist* OR anesthesist* OR cardiologist* OR dermatologist* OR doctor* OR endocrinologist* OR gastroenterologist* OR "general practitioner*" OR geriatrician* OR geriatrist* OR gerontologist* OR gynaecologist* OR gynecologist* OR hepatologist* OR hospitalist* OR "house staff*" OR intensivist* OR intern OR internist* OR interns OR internship* OR "medical speciali*" OR neonatologist* OR nephrologist* OR neurologist* OR neurosurgeon* OR obstetrician* OR obstaetrician* OR oncologist* OR ophthalmologist* OR orthopedist* OR osteopath OR osteopaths OR otolaryngologist* OR otologist* OR paediatrician* OR pathologist* OR pediatrician* OR physiatrist* OR physician* OR pulmonologist* OR radiologist* OR residencies OR residency OR resident* OR resident OR residents OR rheumatologist* OR surgeon* OR urologist*) OR AB(allergist* OR anaesthesiologist* OR anaesthesist* OR anesthesiologist* OR anesthesist* OR cardiologist* OR dermatologist* OR doctor* OR endocrinologist* OR gastroenterologist* OR "general practitioner*" OR geriatrician* OR geriatrist* OR gerontologist* OR gynaecologist* OR gynecologist* OR hepatologist* OR hospitalist* OR "house staff*" OR intensivist* OR intern OR internist* OR interns OR internship* OR "medical speciali*" OR neonatologist* OR nephrologist* OR neurologist* OR neurosurgeon* OR obstetrician* OR obstaetrician* OR oncologist* OR ophthalmologist* OR orthopedist* OR osteopath OR osteopaths OR otolaryngologist* OR otologist* OR paediatrician* OR pathologist* OR pediatrician* OR physiatrist* OR physician* OR pulmonologist* OR radiologist* OR residencies OR residency OR resident* OR resident OR residents OR rheumatologist* OR surgeon* OR urologist*) OR KW(allergist* OR anaesthesiologist* OR anaesthesist* OR anesthesiologist* OR anesthesist* OR cardiologist* OR dermatologist* OR doctor* OR endocrinologist* OR gastroenterologist* OR "general practitioner*" OR geriatrician* OR geriatrist* OR gerontologist* OR gynaecologist* OR gynecologist* OR hepatologist* OR hospitalist* OR "house staff*" OR intensivist* OR intern OR internist* OR interns OR internship*	**474,914**

	OR “medical speciali*” OR neonatologist* OR nephrologist* OR neurologist* OR neurosurgeon* OR obstetrician* OR obstaetrician* OR oncologist* OR ophthalmologist* OR orthopedist* OR osteopath OR osteopaths OR otolaryngologist* OR otologist* OR paediatrician* OR pathologist* OR pediatrician* OR physiatrist* OR physician* OR pulmonologist* OR radiologist* OR residencies OR residency OR resident* OR resident OR residents OR rheumatologist* OR surgeon* OR urologist*)	
**S3**	S1 AND S2	**2,191**
**S2**	MH "Nurses+" OR MH "Nurses by Educational Level+" OR MH "Nurses by Role+" OR MH "Pediatric Nurse Practitioners+" OR MH "Nurse Practitioners+" OR MH "Advanced Practice Nurses+" OR MH "Nurse Administrators+" OR MH "Nurse Managers+" OR MH "Nurse Consultants+" OR MH "Nurses by Specialty+" OR MH "Nurses, Other+" OR MH "Nursing Manpower+" OR MH "Nurse-Patient Relations" OR MH "Academy of Neonatal Nursing" OR MH "Addictions Nursing" OR MH "Advanced Nursing Practice" OR MH "Nursing Administration Research" OR MH "Specialties, Nursing+" OR MH "Community Health Nursing+" OR MH "Emergency Nursing+" OR MH "Maternal-Child Nursing+" OR MH "Neonatal Nursing+" OR MH "Pediatric Critical Care Nursing+" OR MH "Pediatric Nursing+" OR MH "Medical-Surgical Nursing+" OR MH "Dermatology Nursing+" OR MH "Critical Care Nursing+" OR MH "Cardiovascular Nursing+" OR MH "Gerontologic Nursing+" OR MH "Neuroscience Nursing+" OR MH "Oncologic Nursing+" OR MH "Surgical Nursing, Plastic+" OR MH "Wound, Ostomy and Continence Nursing+" OR MH "Psychiatric Nursing+" OR MH "Nursing Care" OR MH "Schools, Nursing" OR MH "Students, Nursing+" OR MH "Students, Nursing, Baccalaureate+" OR MH "Students, Nursing, Graduate+" OR MH "Students, Nursing, Practical" OR MH "Nursing Assistants" OR MH "Nursing Organizations+" OR MH "Nursing Organizations, International+" OR MH "State Boards of Nursing+" OR MH "State, Provincial and Territorial Nursing Organizations+" OR MH "State Nursing Organizations+" OR MH "Nursing Informatics" OR MH "Nursing Theory+" OR MH "Nursing Models, Theoretical+" OR MH "King Open Systems Model+" OR MH "Education, Nursing, Research-Based" OR MH "Clinical Nursing Research" OR MH "Research, Nursing" OR MH "Nursing Staff, Hospital" OR TI(nurse OR nurses OR nursing*) OR AB(nurse OR nurses OR nursing*) OR KW(nurse OR nurses OR nursing*)	**832,526**
**S1**	MH "Diagnostic Reasoning" OR (MH "Judgment" AND (TI(clinical) OR AB(clinical) OR KW(clinical)) OR TI (“clinical reasoning*” OR “clinical judgement*” OR “clinical judgment*” OR “collaborative reasoning*”) OR AB (“clinical reasoning*” OR “clinical judgement*” OR “clinical judgment*” OR “collaborative reasoning*”) OR KW (“clinical reasoning*” OR “clinical judgement*” OR “clinical judgment*” OR “collaborative reasoning*”)	**8,089**

Ebsco/APA PsycInfo May 1, 2020 (968 nurse, 1774 doctor)

Search ID#	Search Terms	Actions
**S8**	S6 NOT *[2501 PMIDs]*	**1,500**
**S7**	S3 NOT *[1415 PMIDs]*	**738**
**S6**	S5 NOT S3	**1,774**
**S5**	S1 AND S4	**1,965**
**S4**	DE "Physicians" OR DE "Family Physicians" OR DE "General Practitioners" OR DE "Gynecologists" OR DE "Internists" OR DE "Neurologists" OR DE "Obstetricians" OR DE "Pathologists" OR DE "Pediatricians" OR DE "Psychiatrists" OR DE "Surgeons" OR TI(allergist* OR anaesthesiologist* OR anaesthesist* OR anesthesiologist* OR anesthesist* OR cardiologist* OR dermatologist* OR doctor* OR endocrinologist* OR gastroenterologist* OR “general practitioner*” OR geriatrician* OR geriatrist* OR gerontologist* OR ...	**207,742**
**S3**	S1 AND S2	**968**
**S2**	DE "Nurses" OR DE "Psychiatric Nurses" OR DE "Public Health Service Nurses" OR DE "School Nurses" OR DE "Nursing" OR DE "Nursing Students" OR DE "Nursing Education" OR TI(nurse OR	**97,210**

	nurses OR nursing*) OR AB(nurse OR nurses OR nursing*) OR KW(nurse OR nurses OR nursing*)	
**S1**	DE "Inductive Deductive Reasoning" OR DE "Reasoning" OR DE "Inference" OR DE "Clinical Judgment (Not Diagnosis)" OR DE "Probability Judgment" OR (DE "Judgment" AND (TI(clinical) OR AB(clinical) OR KW(clinical)) OR TI("clinical reasoning*" OR "clinical judgement*" OR "clinical judgment*" OR "collaborative reasoning*") OR AB("clinical reasoning*" OR "clinical judgement*" OR "clinical judgment*" OR "collaborative reasoning*") OR KW("clinical reasoning*" OR "clinical judgement*" OR "clinical judgmen ...	**36,164**

Clarivate Analytics/Web of Science Core Collection 27 May 27, 2020 (1123 nurse, 2174 doctor)

Set	Results	Query
**#8**	**461**	#7 NOT *[2501 PMIDs]*
**#7**	**2,174**	#6 NOT #3
**#6**	**2,383**	#1 AND #5
**#5**	**1,091,016**	TOPIC: ("allergist*" OR "anaesthesiologist*" OR "anaesthesist*" OR "anesthesiologist*" OR "anesthesist*" OR "cardiologist*" OR "dermatologist*" OR "doctor*" OR "endocrinologist*" OR "gastroenterologist*" OR "general practitioner*" OR "geriatrician*" OR "geriatrist*" OR "gerontologist*" OR "gynaecologist*" OR "gynecologist*" OR "hepatologist*" OR "hospitalist*" OR "house staff*" OR "intensivist*" OR "intern" OR "internist*" OR "interns" OR "internship*" OR "medical speciali*" OR "neonatologist*" OR "nephrologist*" OR "neurologist*" OR "neurosurgeon*" OR "obstetrician*" OR "obstaetrician*" OR "oncologist*" OR "ophthalmologist*" OR "orthopedist*" OR "osteopath" OR "osteopaths" OR "otolaryngologist*" OR "otologist*" OR "paediatrician*" OR "pathologist*" OR "pediatrician*" OR "physiatrist*" OR "physician*" OR "pulmonologist*" OR "radiologist*" OR "residencies" OR "residency" OR "resident*" OR "resident" OR "residents" OR "rheumatologist*" OR "surgeon*" OR "urologist*")
**#4**	**337**	#3 NOT *[1415 PMIDs]*
**#3**	**1,123**	#2 AND #1
**#2**	**286,740**	TOPIC: ("nurse" OR "nurses" OR "nursing*")
**#1**	**9,377**	TOPIC: ("clinical reasoning*" OR "clinical judgement*" OR "clinical judgment*" OR "collaborative reasoning*")
