# Supplementary material for: Reasoning like a doctor or like a nurse? A systematic integrative review
Source: Front Med (Lausanne). 2023 Mar 3;10:1017783. doi: 10.3389/fmed.2023.1017783 (PMC10020202; doi:10.3389/fmed.2023.1017783)
Supplement: Supplementary file 2 [file Data_Sheet_2.pdf]

Appendix 2 Data synthesis

Validity tables of onion shells, condensed

The blue cells contain findings that were common to the two professions and the pink cells contain findings that were in contrast between the two professions. The red data elements in the cells were found only in either nursing or medical literature.

Medicine		Nursing
**Professional paradigms**		
Based on 24 studies		
'doing the doctor thing' (diagnose and treatment) [1]<br>Hippocratic or medical healthcare paradigm: diagnosis, treatment, privacy [2]<br>To manage diagnostic uncertainty [3]<br>The healing relationship between patient and physician [4]	**Cure**	To execute residents' decisions, to indicate observations and concerns [5]
A call for a shift in clinical care away from underlying disease pathology [6]<br>Functional health paradigm [2]<br>Primary care has a broad, generalist scope with a focus on continuity and recognizes psychosocial factors in process and outcome of health problems [7]<br>Pragmatic approach [8]	**Care**	'Doing the nurse thing': attending to patient needs [1]<br>Attitudes and philosophy of encompassed caring [9-12], directed to the concerns of the individual, family or community client system [13]<br>Nurse are not involved in reaching a diagnosis or making management decisions, according to residents [5]<br>Primary care and total client care [14, 15]<br>The importance of comfort [16]<br>The responses of persons, groups or communities toward health problems or vital processes [17]<br>Pragmatism paradigm [18]
Popperian, analytical approach [8, 19]<br>3rd person knowing, objective, generalizable, abstracted from context [6, 20]<br>Clinicians are Bayesians by nature [21]<br>Is the body a machine?[22]<br>Epistemic approach [8]<br>Biomedical, disease model [6, 7, 23]<br>Scientific rigour [19], Evidence based approach [23]	**Objective**	Positivist approach does not fit nursing [15]<br>Empirical paradigm [18]
Understanding people and their experience of disease [6]<br>Second person knowing, intersubjectivity, links objective and subjective [6]<br>Situated clinical knowledge, temporary, relative and individualized [24]<br>Medical practice is a hermeneutical enterprise [24]<br>Is the physician a neutral observer? [20]<br>Clinical medicine is a relationship between two individuals [4]<br>psychosocial illness model [7, 23]<br>Clinical relevance [19], medical practice is not the same as medical science [22, 24]<br>Act for the individual patient [4]	**Subjective**	Interpretive paradigm [18]<br>The client's and his/her own perceptions [13]<br>The intention to humanize and personalize care [16]<br>Care requires interpretation, understanding and hermeneutic experience [12]<br>Commitment, empowerment, self-awareness and holism [9]<br>Patient centered care [1, 10]
Patient as a partner, or passive [23]<br>Privacy contract between patient and physician [2]<br>Patient not necessarily involved in clinical reasoning [14]<br>Intersubjectivity [6]	**Relation to patient**	The ethic for disclosure to patients and families [16]<br>Patient is involved in reasoning [14]

Medicine		Nursing
**Underpinning theories**		
Based on 36 studies		
Information processing theory [8, 25-28]<br>Script or schema theory [27, 29, 30]<br>Exemplar theory [31, 32] Prototype theory [31, 32] Fuzzy trace theory [19],	**Memory and cognition**	Information processing theory [10, 11, 26, 33-37]. Lee et al [15] question the value of this theory not only logic, objectivity and rationality but also emotions, affect and context)<br>Script or schema theory [11, 33]
Dual (cognition) process theory [6, 19, 21, 26-28, 30, 38-42]<br>Cognitive Continuum theory [19, 21, 43]<br>Skills acquisition theory Dreyfus [21, 28]<br>Bounded rationality [41, 44]<br>Theory of heuristic strategies [19]	**Rationality, intuition and analysis,**	Dual (cognition) process theory [26, 45]<br>Cognitive Continuum theory [43, 46-48]<br>Skills acquisition theory Dreyfus [33]<br>Dreyfus and Dreyfus model of intuitive judgment [15, 33]
Situativity theory [25, 27] Cognitive load theory [25, 27], Dual Coding theory of Paivio [49], Gallagher's interactional theory [6], virtue epistemology [6], belief formation theory, [6] Lonergan's theory of knowing, [24] Dialogism [22]	**Perception and interaction**	Situativity theory [50]<br>Schon's reflection on action [9], Social judgment theory [47]

Medicine		Nursing
**Intentions of clinical reasoning**		
Based on 34 studies		
To establish a diagnosis [5, 6, 14, 25, 27, 30, 34, 38, 41, 51, 52] with a strong focus on etiology [5], biological alterations [14]<br>Problem construction [8]<br>To predict [14, 42]	**To diagnose or assess**	To establish or identify a diagnosis [5]<br>actual and potential patient problems [10, 26, 33, 34, 37, 50]<br>Problem construction of current (constantly changing) condition or situation [26, 53]
To decide on a plan of actions [5, 8, 22, 25, 27, 51, 54]<br>Admission decisions [38]<br>Treatment [6, 14, 26, 30, 41, 42, 55]<br>To prevent [39]<br>To monitor the patient [5]	**Patient management**	To decide on a plan of actions [5, 36, 37, 48, 54]<br>To prevent [13, 33, 50]<br>To monitor the patient [5]
To explain to the patient [5, 14, 26, 53]<br>To understand patient's problems [6, 20]	**To understand and explain**	To explain to the patient [5]<br>To understand the symptom and its impact on the patient [5, 37]
To reduce uncertainty [3, 56]<br>To develop hypotheses and theories [8, 42]	**To enlarge knowledge**	To manage uncertainty [57]<br>To improve competent nursing practice [36, 45, 58]
To communicate interprofessionally and reach a shared mental model about patients problem and management [5]	**To collaborate**	To communicate interprofessionally and reach a shared mental model about patients problem and management [5]<br>To promote nursing autonomy [45]
To improve patient outcomes [6, 38, 39]<br>To improve care [59]	**To achieve**	To improve patient outcomes[13, 33, 34, 36, 37, 45, 48, 54, 57]<br>To improve care [45, 48])
Framing the encounter[51]	**To frame**	

Medicine		Nursing
**What, content, domain**		
Based 31 studies		
Disease, illness, problems, symptoms, progress, pathophysiology [6, 14, 15, 19-21, 25, 26, 28, 43, 60]<br>Complaints [8, 24]<br>Health, appearance, environment [38, 44]<br>*"The doctor must come to know the patient's body as a biomedical object, but must also reach an understanding with the patient as a fellow human being…Both are necessary and both should be used in balance"*[22]	**focus**	Disease, illness, problems, symptoms, progress, pathophysiology [16, 26, 50, 58]<br>Needs, concerns, emotions and feelings [1, 5, 13, 45, 48]<br>Clinical situation, health status, strengths and resources, psychosocial condition [5, 13, 15, 16, 26, 34, 45, 54, 58, 61]<br>Responses to health problems, risks [5, 16, 50, 61]
	**Clientele**	Also client system, community [13, 14, 16, 26]

Medicine		Nursing
	**Antecedents of clinical reasoning**	
Based on 54 studies		
Clinical experience [5, 21, 25, 27, 28, 39, 40, 52, 55, 60, 62, 63]	**Professional experience**	Clinical experience [5, 9, 11, 15, 16, 33, 34, 36, 37, 48, 54, 64], The role of experience is not fully understood [45, 64]
Knowledge [5, 21, 27, 29, 30, 49, 59, 63]<br>Formal knowledge of biomedicine, epidemiology, social and human sciences, of signs, symptoms, causes, pathophysiological mechanisms, treatments, drugs, of local services [20, 21, 39, 42, 55, 63]<br>Encapsulated knowledge [21, 31, 59]<br>Domain or discipline specific knowledge [32, 40]<br>Informal, tacit knowledge [5, 20-22, 39]<br>Organized knowledge (patterns, scripts) [25, 28-30, 32, 42, 55, 62]<br>Physicians use more theoretical knowledge [26]	**Knowledge**	Knowledge [5, 11, 15, 34, 54], integrative knowledge from different areas [45],<br>Formal knowledge of physiology, pathophysiology, pharmacology, epidemiology, therapeutics, culture, context of care, ethics and law etc. as well as an understanding of evidence based practice [11, 16, 33, 34, 45, 48, 50, 57, 58, 64]<br>Encapsulated knowledge [61]<br>Domain or discipline specific knowledge [15, 34, 36, 37, 57, 61]<br>Informal, tacit knowledge [5, 9, 11, 16, 34]<br>Organized knowledge (patterns, scripts)[9-11, 58]<br>Procedural knowledge [33, 34, 48]<br>Nurses use more personal knowledge [26]
(early) Patient data [8, 20, 30, 38, 40], contextual data [20, 41]<br>Diagnostic uncertainty [38]	**Trigger**	(early) Patient data [10, 16, 34, 35, 50, 54, 57]<br>Patient's needs [11]
Task characteristics, complexity [19, 21, 28, 32, 40]<br>Characteristics of the illness: [5, 19, 25, 39, 42, 52, 63]	**Characteristics task**	Task characteristics, complexity [15, 16, 34, 47]<br>Characteristics of the situation [9, 34]
Metacognition and logic [8, 19, 20, 24, 30, 42, 62],<br>Intuition [20]<br>Memory and retrieval [32]<br>Perception and situational awareness [5, 6, 52, 56, 60], alertness [60]<br>**Personal**: Emotions [6, 21, 55, 60, 65], affect [21], Motivation [30, 56, 60], Level of confidence [5, 44], Self-knowledge [6], Age, sex, training, beliefs, fear of litigation, risk aversion [40], ability to deal with diagnostic uncertainty [39, 40], cognitive load [19, 25, 40, 56], general approach, as an interventionist or gradualist [43] emotional intelligence [65], mastery of rhetoric and language [22]	**Characteristics professional**	Metacognition and logic [11, 16, 33, 34, 36]<br>Intuition [11, 35, 54]<br>Memory and retrieval [33, 34, 54]<br>Perception and situational awareness [5, 15, 33, 34], Sense of salience [11, 33, 35, 57]<br>**Personal,** "*What the nurse brings to the situation*" [16, 57, 58] general approach, as an interventionist or gradualist [43] a questioning attitude [13, 17], the ability to manage trust, knowledge and skills simultaneously in the situation with the patient [12] attention, concentration, motivation [13, 45]
Rapport with patient [24, 27, 39, 52, 55], intersubjectivity [6]<br>Knowing the patient [5, 6]<br>Team [5]	**Relations**	Rapport with patient [9, 15, 37, 48, 54, 58, 64, 66], mutual trust [12, 43]<br>Knowing the patient [5, 9, 15, 16, 43, 58, 64]<br>Team [5] (Interdisciplinary) relationships power, inequity, role expectations [15, 16, 33]

Medicine		Nursing
**Attributes and strategies of clinical reasoning**		
Based on 55 studies		
**Recognition** [19], Similarity recognition, [31, 59]. pattern recognition on few cues [19, 21, 24, 27, 31, 38, 42, 43, 52, 55, 62], based on specific instances or general prototypes [32]<br>Recognized patterns are followed by guided search using your slower, sequential reasoning brain to match patterns [60]<br>**Perception** of the features of the situation that trigger scripts [29], to notice and to discriminate between right and wrong and (un)similarity. Perception and cognition are integrated [60]<br>**Memory** direct retrieval by categorization and problem representation [31, 55] Scripts and schemes, [25, 29, 42]<br>**Intuition** [19, 43, 60, 62], first impressions [40], instantaneous formulations [39], intuitive knowing is based on experience, understanding and judging [24], bounded rationality [19]<br>**Interaction of intuitive and analytical cognition** [6, 19, 22, 24, 27, 38, 52]<br>**Reflection** [24]	**Use of cognition: memory, perception, attention, recognition, intuition**	**Recognition** [9, 10, 17, 45], similarity recognition [18, 33, 64], pattern recognition [10, 11, 16, 33, 34, 37, 43, 47, 57], based on instances or general types [16]<br>**Perception** noticing [16, 45, 58]<br>**Memory**, scripts [11] information processing [34], recall knowledge [33, 50]<br>**Intuition** [34]**,** quasi rationality [47]<br>**Interaction of intuitive and analytical cognition** [11, 45, 47, 48, 50, 58], cognition and metacognition [54, 58]<br>**Reflection** [16, 58]
**Assessment** Starting point of reasoning: signs and symptoms [5, 56, 62] to clarify issues and priorities [39, 41], to detect changes [5] environmental cues [44], pre-reflexive experiences and imaginations of the GP related to the patient's presentations guide assessment [24], environment and interaction are true variance [27]<br>**Risk** assessment [30, 39]<br>**Interpretation and integration of data** [8, 43, 56, 62] from complaint to solution [8], through interaction and perception [24], connecting similarity with causal explanations [60], chunking data by semantic qualifiers [27], inferring from pattern to illness script [60]. To describe data in verbally in logical, causal explanations [60] into clinical vocabulary [67]<br>Use probability thresholds [19, 30]	**Data analysis and interpretation**	**Assessment** Search and evaluate clinical and other data [5, 10, 11, 34, 37, 48] of the patient and the environment [34], to detect changes, feelings, emotions and general condition of the patient [5], to identify cues, diagnoses, re-evaluate data, identifying missing information[35]<br>**risk f**actors [35], distinguishing normal form abnormal and relevant form irrelevant data [35, 48]<br>**Interpretation and integration of data** [10, 16, 35, 43, 45, 47, 48, 50, 54, 57, 58], intentional judgment [17, 35, 45], forming relationships (assertions) [37], framing [11], with simultaneously planning interventions [36] problems and interventions are viewed as linked entities [33]
**Hypothetical reasoning**, formulating hypotheses [5, 21] and subsequent paths for elaboration [20] initial set of working hypotheses [14]<br>**Hypothetico-deductive reasoning** [5, 19, 27, 28, 34, 38, 43, 52, 55, 60]<br>**Hypothesis generation** (divergence of hypotheses) [56] , by intuitive processes [28, 29, 40]<br>**Hypothesis testing** [21, 24] a strategy of confirming hypotheses [32, 44], ruling out serious illnesses, ruling in common and safe conditions [29, 39]	**Generation of hypothesis or alternatives**	**Hypothetico-deductive reasoning**, [9, 10, 16, 43]<br>**Hypothesis generation**, [11, 48, 54, 61], early [9], by hypothetico abductive reasoning (to generate specific theories to explain individual cases from our own, personal tacit knowledge about those cases.' [9] Generation of alternatives [54]<br>**Hypothesis testing** [18] testing predictions [37]<br>**Formulating hypotheses** [5, 10, 34]. These are hypothesis –candidates (because they are without some form of causality or predictive power) [14], identifying assumptions [35, 37]
**Logic** Inferential methods of induction, deduction and abduction [6], to find the best explanation or justification [14], an inductive approach is effective for exploratory tasks that do not have distinct goals, while a deductive approach is more useful for diagnostic and classification tasks [42]<br>Inductive/forward, from current to ideal state is related to system 1 strategies [19, 42], deductive/backwards, from desired result to current situation, a goal-drive approach, testing hypothesis, related to system 2. [19, 42], to apply general principles to specific cases [32], Malterud calls this practical reasoning [20]<br>Abduction in the context of justification [14]<br>**Heuristics** and mental shortcuts**,** [19, 24, 28], simple, general and hypothesis specific heuristics, anchor [44] or affect heuristic [21] gestalt effect [19]	**Cognitive strategies, heuristics, inferences, approaches**	**Logic** [34], deliberate rationality [33], making inferences [17, 35, 45, 47, 54, 64] , induction and deduction [34, 35, 37, 61], deduction**,** when testing hypotheses [18], induction [10, 11], to simultaneously assemble and evaluate patient information and supportive evidence [11], to choose management [18]<br>Abduction, inference to the best explanation inferring causal mechanisms, and hypothesis generation [18] in the context of discovery [14], cause and effect assertions [11]<br>**Heuristics** [9-11, 34, 37, 54]**,** to speed reasoning processes [36], or to estimate probability [61] rule of thumb, availability, anchoring [10], clinical Grasp [57] common sense understanding [33]<br>**Nonanalytic, system 1 strategies**

**Nonanalytic, system 1 strategies** [30, 39], in the initial stage of the encounter [55]<br>pattern matching [28, 32], gut feelings*, often limited to prognostic assessments of the patient's situation and are often accompanied by bodily sensations, a sense of alarm and a sense of reassurance* [21]*, gut feelings based on interaction between patient information and GP's knowledge and experience,* intersubjectivity [6], experiential-inductive approach, modular responsivity [19]<br>**Analytic, system 2 strategies** [6, 27, 30, 39] abstract, decontextualized, and rational approach to clinical problem-solving [6], categorization [6, 31, 32], problem construction [8], aetiology-based reasoning [5], causal reasoning [21]<br>**Approaches**<br>To generate meaning through interaction [22, 38, 39]<br>Checking accuracy of mental representations [41]<br>Backwards or forwards reasoning [29, 39]<br>Reflective diagnostic time out [38], Distraction [38]<br>Relational strategies: analogy (similarity), anomaly (discrepancy/unusualness), antinomy (incompatibility), antithesis (opposition), as a metacognitive means to switch between system 1 and 2 thinking [30]<br>Bayesian thinking [19, 30]<br>Error-checking strategies, to think of other possibilities or interpretations of data [31]<br>Checkpoint strategies, with a focus on red flags [38, 39]<br>therapeutic reasoning [55]		Informal thinking strategies: setting priorities, making generalizations [34], narrative thinking, routine thinking [64], fast and frugal reasoning [68], pattern matching, empathizing, developing a bigger picture and balancing preferences with differences [9], intuitive clinical reasoning [9, 10, 16, 43, 47, 48, 57, 64]. Intuition is a function of experience [61] and difficult to verbalize and explain [50]<br>**Analytical, system 2 strategies** [33, 45, 47, 50, 57, 64]**,** to link cues to categories, classification process, with a probabilistic relationship between cues and class, as an exemplar or a prototype [11, 36, 37, 61], theoretical reasoning [11]<br>**Approaches**<br>Diagnostic reasoning [11, 16]**,** problematic reasoning [11]<br>deliberation [54]**,** pondering [37] , operational reasoning [11]<br>backwards reasoning [46]<br>Narrative thinking, trying to understand the particular [16]<br>Predictive reasoning [9]<br>Responding, acting [16, 58]<br>Making personal connection [37]
Non-linear [24, 27, 38, 55]<br>Context-specific and case specific [27]<br>As an individual or collaborative process to communicate and to share and integrate knowledge [56]<br>As a process [24, 38, 51, 56, 69]	**Process**	Non-linear [33-35, 48, 50]<br>Context-dependent and domain-specific [33, 34]<br>Reactive or proactive [37]<br>As a Process [16, 33, 35, 36, 50, 58]

Medicine		Nursing
**Outcomes**		
Based on 27 studies		
Diagnosis [1, 21, 24, 27, 56, 62], prioritized problem list, differential diagnosis [25]	**Diagnosis**	Diagnosis of actual and potential problems [35]empirical generalizations [14]<br>clinical diagnoses [5], an accurate picture of current condition or situation [53]
Clinical decisions [39, 40], decisions about discharge, admission [38, 40], diagnostic disclosure [39, 44]	**Decisions**	Clinical decisions [33, 34, 37, 48, 54, 57, 61], decision to alert residents [5]
Treatment or management plan [8, 14, 21, 25, 27, 40, 56, 63], Referrals [52, 63]	**Management**	Choice of action [34, 45, 54, 57, 58], care plan [5, 10, 35]
Prognosis [14, 21, 63]	**Prognosis**	Prognosis [35, 37]
Judgement of the urgency of approach [38, 41]	**Judgment**	Judgement [34, 37, 54], of degree of urgency [5]
Team effectiveness [5]	**Collaboration**	Team effectiveness [5]
To provide explanations to patients to confirm the management plans [5]	**Explanations**	To provide explanations to patients about procedures [5, 37]<br>Justification for decisions [45]
	**New knowledge**	Reflection and learning [57]

Medicine		Nursing
**Contextual factors**		
Based on 22 studies		
Context of the patient [21, 44]<br>Patient expectations and preferences [40, 52, 63]<br>Patient characteristics [19, 25, 40, 63]<br>Relationship and barriers [19, 25, 40, 63]	**Patient-related factors**	Context of the patient [34]<br>Patient characteristics [35]
Care context and work environment [19, 27, 32, 40, 51]<br>Resources and legal issues [19, 40, 52, 63]<br>Time aspects and workload [19, 25, 38, 40, 56]	**Environment-related factors**	Care context and work environment [9, 11, 16, 34, 35, 58]<br>Culture and social context of the unit/team [16, 33, 58, 61, 64]<br>Time aspects and workload [9, 34, 64, 68]

References

1. McLean, M., *From being a nurse to becoming a 'different' doctor.* Adv Health Sci Educ Theory Pract, 2017. **22**(3): p. 667-689.
2. Franco, A.A., H. Bouma, and J.E.M.H.V. Bronswijk, *Health care paradigms in transition.* Gerontechnology, 2014. **13**(1).
3. Alam, R., et al., *Managing diagnostic uncertainty in primary care: a systematic critical review.* BMC Family Practice, 2017. **18**: p. 1-13.
4. Davis, F.D., *Phronesis, clinical reasoning, and Pellegrino's philosophy of medicine.* Theor Med, 1997. **18**(1): p. 173-95.
5. Blondon, K.S., et al., *Interprofessional collaborative reasoning by residents and nurses in internal medicine: Evidence from a simulation study.* Medical Teacher, 2017. **39**(4): p. 360-367.
6. Gupta, M., N. Potter, and S. Goyer, *Diagnostic Reasoning in Psychiatry: Acknowledging an Explicit Role for Intersubjective Knowing.* Philosophy Psychiatry & Psychology, 2019. **26**(1): p. 49-64.
7. Evans, L. and D. Trotter, *Epistemology and Uncertainty in Primary Care: An Exploratory Study.* Family Medicine, 2009. **41**(5): p. 319-325.
8. Pottier, P. and B. Planchon, *[Description of the mental processes occurring during clinical reasoning].* Rev Med Interne, 2011. **32**(6): p. 383-90.
9. Crook, J.A., *How do expert mental health nurses make on-the-spot clinical decisions? A review of the literature.* Journal of Psychiatric and Mental Health Nursing, 2001. **8**(1): p. 1-6.
10. Judd, J., *Strategies used by nurses for decision-making in the paediatric orthopaedic setting.* Journal of Orthopaedic Nursing, 2005. **9**(3): p. 166-171.
11. Banning, M., *Clinical reasoning and its application to nursing: concepts and research studies.* Nurse Education in Practice, 2008. **8**(3): p. 177-83.
12. Austgard, K.I., *What characterises nursing care? A hermeneutical philosophical inquiry.* Scand J Caring Sci, 2008. **22**(2): p. 314-9.
13. Fawcett, J., B. McDowell, and D.M. Newman, *In response to: Simmons (2010) Clinical reasoning: concept analysis. Journal of Advanced Nursing 66 (5), 1151-1158.* J Adv Nurs, 2010. **66**(12): p. 2839-40.
14. Chiffi, D. and R. Zanotti, *Medical and nursing diagnoses: a critical comparison.* Journal of Evaluation in Clinical Practice, 2015. **21**(1): p. 1-6.
15. Lee, J., A.C.M. Chan, and D.R. Phillips, *Diagnostic practise in nursing: a critical review of the literature.* Nursing & Health Sciences, 2006. **8**(1): p. 57-65.
16. Tanner, C.A., *Thinking Like a Nurse: A Research-Based Model of Clinical Judgment in Nursing.* Journal of Nursing Education, 2006. **45**(6): p. 204-211.
17. Passos Vaz da Costa, C. and M.H. Barros Araújo Luz, *Nursing scientific production on diagnostic reasoning: integrative review.* Journal of Nursing UFPE / Revista de Enfermagem UFPE, 2016. **10**(1): p. 152-162.
18. Mirza, N.A., et al., *A concept analysis of abductive reasoning.* J Adv Nurs, 2014. **70**(9): p. 1980-1994.
19. Croskerry, P., *A Universal Model of Diagnostic Reasoning.* Academic Medicine, 2009. **84**(8): p. 1022-1028.
20. Malterud, K., *Reflexivity and metapositions: strategies for appraisal of clinical evidence.* J Eval Clin Pract, 2002. **8**(2): p. 121-6.
21. Stolper, E., et al., *Gut feelings as a third track in general practitioners' diagnostic reasoning.* Journal of General Internal Medicine, 2011. **26**(2): p. 197-203.
22. Loftus, S., *Rethinking clinical reasoning: time for a dialogical turn.* Med Educ, 2012. **46**(12): p. 1174-8.
23. Benbassat, J., *[Paradigmatic shifts in clinical practice in the the last generation].* Harefuah, 1996. **130**(9): p. 585-9, 656.
24. Malterud, K., S. Reventlow, and A.D. Guassora, *Diagnostic knowing in general practice: interpretative action and reflexivity.* Scandinavian Journal of Primary Health Care, 2019. **37**(4): p. 393-401.
25. Durning, S.J., et al., *The impact of selected contextual factors on experts' clinical reasoning performance (does context impact clinical reasoning performance in experts?).* Adv Health Sci Educ Theory Pract, 2012. **17**(1): p. 65-79.
26. Salantera, S., et al., *Clinical judgement and information seeking by nurses and physicians working with cancer patients.* Psychooncology, 2003. **12**(3): p. 280-90.
27. Durning, S.J., et al., *Clarifying assumptions to enhance our understanding and assessment of clinical reasoning.* Acad Med, 2013. **88**(4): p. 442-8.
28. Round, A., *Introduction to clinical reasoning.* Journal of Evaluation in Clinical Practice, 2001. **7**(2): p. 109-117.

29. Charlin, B., J. Tardif, and H.P.A. Boshuizen, *Scripts and medical diagnostic knowledge: Theory and applications for clinical reasoning. Instruction and research.* Academic Medicine 2000. **75**(2): p. 182-90.
30. Dumas, D., D.M. Torre, and S.J. Durning, *Using Relational Reasoning Strategies to Help Improve Clinical Reasoning Practice.* Acad Med, 2018. **93**(5): p. 709-714.
31. Norman, G., M. Young, and L. Brooks, *Non-analytical models of clinical reasoning: the role of experience.* Med Educ, 2007. **41**(12): p. 1140-5.
32. Elstein, A.S. and A. Schwarz, *Clinical problem solving and diagnostic decision making: Selective review of the cognitive literature.* BMJ: British Medical Journal, 2002. **324**(7339): p. 729-732.
33. Holder, A.G., *Clinical Reasoning: A State of the Science Report.* International Journal of Nursing Education Scholarship, 2018. **15**(1): p. 1-10.
34. Simmons, B., *Clinical reasoning: concept analysis.* Journal of Advanced Nursing, 2010. **66**(5): p. 1151-8.
35. Lee, J., et al., *Registered nurses' clinical reasoning skills and reasoning process: A think-aloud study.* Nurse Educ Today, 2016. **46**: p. 75-80.
36. Simmons, B., et al., *Clinical reasoning in experienced nurses.* Western Journal of Nursing Research, 2003. **25**(6): p. 701-19; discussion 720-4.
37. Johnsen, H.M., A. Slettebo, and M. Fossum, *Registered nurses' clinical reasoning in home healthcare clinical practice: A think-aloud study with protocol analysis.* Nurse Educ Today, 2016. **40**: p. 95-100.
38. Adams, E., et al., *Clinical reasoning of junior doctors in emergency medicine: a grounded theory study.* Emerg Med J, 2016. **34**(2): p. 70-75.
39. Balla, J., et al., *Clinical decision making in a high-risk primary care environment: a qualitative study in the UK.* BMJ Open, 2012. **2**: p. e000414.
40. Pelaccia, T., et al., *A Scoping Review of Physicians' Clinical Reasoning in Emergency Departments.* Ann Emerg Med, 2020. **75**(2): p. 206-217.
41. Pelaccia, T., et al., *Insights into emergency physicians' minds in the seconds before and into a patient encounter.* Intern Emerg Med, 2015. **10**(7): p. 865-73.
42. Shin, H.S., *Reasoning processes in clinical reasoning: from the perspective of cognitive psychology.* Korean J Med Educ, 2019. **31**(4): p. 299-308.
43. Taylor, F., *A comparative study examining the decision-making processes of medical and nursing staff in weaning patients from mechanical ventilation.* Intensive and Critical Care Nursing, 2006. **22**(5): p. 253-263.
44. Bonilauri Ferreira, A.P., et al., *Clinical reasoning in the real world is mediated by bounded rationality: implications for diagnostic clinical practice guidelines.* PLoS One, 2010. **5**(4): p. e10265.
45. Quaresma, A., D. Modernel Xavier, and M.R. Cezar-Vaz, *Raciocínio clínico do enfermeiro: uma abordagem segundo a Teoria do Processo Dual.* Revista Enfermagem UERJ, 2019. **27**: p. 1-6.
46. Twycross, A. and L. Powls, *How do children's nurses make clinical decisions? Two preliminary studies.* Journal of Clinical Nursing, 2006. **15**(10): p. 1324-1335.
47. Cader, R., S. Campbell, and D. Watson, *Cognitive Continuum Theory in nursing decision-making.* Journal of Advanced Nursing, 2005. **49**(4): p. 397-405.
48. Καρρά, Β., et al., *Ποιοτική διερεύνηση της διαδικασίας λήψης νοσηλευτικών κλινικών αποφάσεων σε Μονάδες Εντατικής Θεραπείας.* Nursing Care & Research / Nosileia kai Ereuna, 2018(51): p. 124-137.
49. Edwards, J.C., M. Sadoski, and T.K. Burdenski, Jr., *Physicians' reported use of mental images and language in clinical reasoning.* Imagination, Cognition & Personality, 2004. **24**(1): p. 41-49.
50. Levett-Jones, T., et al., *The 'five rights' of clinical reasoning: an educational model to enhance nursing students' ability to identify and manage clinically 'at risk' patients.* Nurse Education Today, 2010. **30**(6): p. 515-20.
51. Goldszmidt, M., J.P. Minda, and G. Bordage, *Developing a unified list of physicians' reasoning tasks during clinical encounters.* Acad Med, 2013. **88**(3): p. 390-7.
52. Yazdani, S., M. Hosseinzadeh, and F. Hosseini, *Models of clinical reasoning with a focus on general practice: A critical review.* J Adv Med Educ Prof, 2017. **5**(4): p. 177-184.
53. Crow, R.A., J. Chase, and D. Lamond, *The cognitive component of nursing assessment: an analysis.* Journal of Advanced Nursing (Wiley-Blackwell), 1995. **22**(2): p. 206-212.
54. Cote, S. and D. St-Cyr Tribble, *[Clinical reasoning in nursing, concept analysis].* Rech Soins Infirm, 2012(111): p. 13-21.
55. Bissessur, S.W., et al., *Therapeutic reasoning: from hiatus to hypothetical model.* J Eval Clin Pract, 2009. **15**(6): p. 985-9.

56. Kiesewetter, J., F. Fischer, and M.R. Fischer, *Collaborative Clinical Reasoning-A Systematic Review of Empirical Studies.* J Contin Educ Health Prof, 2017. **37**(2): p. 123-128.
57. van Graan, A.C., M.J.S. Williams, and M.P. Koen, *Clinical judgement within the South African clinical nursing environment: A concept analysis.* Health SA Gesondheid, 2016. **21**(1): p. 33-45.
58. Victor-Chmil, J., *Critical thinking versus clinical reasoning versus clinical judgment: differential diagnosis.* Nurse Educ, 2013. **38**(1): p. 34-6.
59. Norman, G., *Research in clinical reasoning: past history and current trends.* Medical Education, 2005. **39**(4): p. 418-27.
60. Cox, K., *Perceiving clinical evidence.* Medical Education, 2002. **36**(12): p. 1189-1195.
61. Buckingham, C.D. and A. Adams, *Classifying clinical decision making: interpreting nursing intuition, heuristics and medical diagnosis.* Journal of Advanced Nursing (Wiley-Blackwell), 2000. **32**(4): p. 990-998.
62. Groves, M., et al., *The clinical reasoning characteristics of diagnostic experts.* Med Teach, 2003. **25**(3): p. 308-13.
63. Pomeroy, S.E. and R.P. Cant, *General practitioners' decision to refer patients to dietitians: insight into the clinical reasoning process.* Aust J Prim Health, 2010. **16**(2): p. 147-53.
64. Cappelletti, A., J.K. Engel, and D. Prentice, *Systematic review of clinical judgment and reasoning in nursing.* Journal of Nursing Education, 2014. **53**(8): p. 453-8.
65. Marcum, J.A., *The Role of Emotions in Clinical Reasoning and Decision Making.* Journal of Medicine and Philosophy, 2013. **38**(5): p. 501-519.
66. Psiuk, T., *[Clinical reasoning of nurses in their daily activities. From clinical reasoning to concepts].* Rech Soins Infirm, 1997(51): p. 12-24.
67. Charlin, B., et al., *Clinical reasoning processes: unravelling complexity through graphical representation.* Med Educ, 2012. **46**(5): p. 454-63.
68. Yang, H., C. Thompson, and M. Bland, *Do nurses reason 'adaptively' in time limited situations: the findings of a descriptive regression analysis.* BMC Med Inform Decis Mak, 2014. **14**: p. 96.
69. Juma, S. and M. Goldszmidt, *What physicians reason about during admission case review.* Adv Health Sci Educ Theory Pract, 2017. **22**(3): p. 691-711.
